# Genome edited animals: Learning from GM crops?

**DOI:** 10.1007/s11248-017-0017-2

**Published:** 2017-04-21

**Authors:** Ann Bruce

**Affiliations:** Science, Technology and Innovation Studies, The University of Edinburgh, Old Surgeons’ Hall, High School Yards, Edinburgh, UK

**Keywords:** Genome edited livestock, Regulation, Production systems, Industry structure, Veterinarians

## Abstract

Genome editing of livestock is poised to become commercial reality, yet questions remain as to appropriate regulation, potential impact on the industry sector and public acceptability of products. This paper looks at how genome editing of livestock has attempted to learn some of the lessons from commercialisation of GM crops, and takes a systemic approach to explore some of the complexity and ambiguity in incorporating genome edited animals in a food production system. Current applications of genome editing are considered, viewed from the perspective of past technological applications. The question of what is genome editing, and can it be considered natural is examined. The implications of regulation on development of different sectors of livestock production systems are studied, with a particular focus on the veterinary sector. From an EU perspective, regulation of genome edited animals, although not necessarily the same as for GM crops, is advocated from a number of different perspectives. This paper aims to open up new avenues of research on genome edited animals, extending from the current primary focus on science and regulation, to engage with a wider-range of food system actors.

## Introduction

Genome editing of livestock is advancing rapidly as a technical field and may be poised to become commercial reality. Yet questions remain as to appropriate regulation, potential impact on the industry sector and public acceptability of products. This paper examines some of the complexity and ambiguity in incorporating genome edited animals into a food production system. It takes as its starting point the commercial introduction of genetically modified crops, and some of the issues raised by early applications of genetic modification to crops.

The commercial introduction of genetically modified (GM) crops in the early 1990s was initially fairly uncontroversial. The widespread introduction in maize, soya and oilseed rape, however, resulted in social campaigns against GM crops in Europe and elsewhere. Nevertheless, annual surveys from ISAAA (2015) indicate widespread adoption of a limited number of modified crops species (mostly oilseed rape, soya and maize) and a limited number of traits (mostly herbicide tolerance and insect resistance). The research pipeline continues to offer novel crop applications (Parisi et al. 2016), although their penetration into practical use has been slow to date. In contrast, there are no genetically modified (GM) food animals being sold for food at the time of writing, to my knowledge.

Several recent developments look set to change this picture. The advent of genome editing techniques, especially CRISPR-Cas9, is enabling changes to be made at the DNA level, much more easily, precisely and cheaply. The recent approvals of genetically modified salmon for consumption both by the U.S. Food and Drug Administration (2015), and Health Canada (2016) provide evidence that it is possible for GM products to satisfy regulatory requirements. In addition, cheaper and faster DNA sequencing is leading to increased understanding of the underlying genetic basis of traits. Together these developments potentially open up new opportunities for marketable genetic modification in food animal production traits, hitherto unachievable.

Technological advances on their own are insufficient for market penetration, however. An entire production system is involved, encompassing breeders, producers, different markets, retailers and consumers as well as regulators, animal welfare advocates, veterinarians and other pertinent stakeholders. And, most critically, products have to be acceptable to publics. A livestock production system thus consists of multiple stakeholders, each adapting to shifting circumstances and opportunities in different ways, but interacting with each other, and affecting each other. These stakeholders each respond to scientific developments, adjustments in markets, changes in methods of production, and economic and social pressures, in their own ways. The result is a kaleidoscope of multiple complex interactions. This paper explores some of this complexity and implications of adoption of genome edited livestock. It does this in the context of early developments in GM crops and their regulation.

This paper draws on long-term observation of developments in genetics and livestock (e.g. Bruce and Bruce 1998), including scientific developments, regulatory changes, ethical and social challenges and industry action. Evidence is based on publications (including grey literature) and attendance at scientific, regulatory and industry meetings, as well as formal and informal conversations with individual stakeholders in livestock production systems. Rapidly developing new technologies are offering novel possibilities that could be attractive to the livestock production sector, making an investigation of this subject timely. This paper seeks to extend the recent focus of publications on regulation of genome edited livestock to a more systems-oriented approach, and suggests that further investigation of interactions among the stakeholders involved in livestock production is therefore important.

This exploration of complexity starts with a review of the current possible applications of genome editing to livestock, based on publicly available data. It shows how choices made by scientists regarding applications to be developed have responded to criticisms of the early developments in GM crops. Having established what kind of genome edited products might be in the pipeline, the next question considered is how these developments relate to some of the key issues which could affect their acceptance. These include arguments around naturalness, transgenesis and regulation that were raised by GM crops. Here I reflect on both the similarities and differences between genetic modification and genome editing. Regulation will also affect the relative competitiveness of different types of companies; who might be the winners and who the losers? The introduction of genome editing will impact on other sectors of the livestock production system. I will focus particularly on interactions with veterinarians, given that many applications of genome editing currently relate to disease resistance. Finally, I reflect on the broader challenges to livestock production and how genome editing relates to these challenges. The perspective will be European, and more specifically from the UK.

## Possibilities of genome edited animals

Animals produced by genome editing offer the promise of precision modification to DNA, which older methods also offered, but in some respects failed to deliver. New precision engineering of the genome (Fahrenkrug et al. 2010) is in contrast to the traditional GM methods that largely relied on transfer of genes between species (Clark and Whitelaw 2003). Early enthusiasm at the prospects from GM animals (e.g. Bulfield 1990) dissipated as producing commercial GM animals proved to be more challenging than seemed at first. A number of developments in molecular biology have taken place over the last 15 year, including gene manipulation, methods of introducing genetic changes into animals, and understanding the underlying genetics (Bruce et al. 2013; Laible et al. 2015; Tan et al. 2016). In particular, the introduction of genome editing tools have revitalised the research area.

Genome editing involves the use of molecular ‘scissors’ to introduce changes into existing DNA, as opposed to classical genetic modification which often involved moving genes from one species to another. Genome editing also enables a much wider-range of changes, for example, gene knock-out, base pair substitution, targeted insertion/deletion of larger genomic regions, and modulation of gene expression (e.g. Tan et al. 2016; Van Eenennaam 2017).

### Social pressures to produce real benefits

In selecting which applications to focus on, many of the developers of genome edited animals have sought to learn from the controversies surrounding GM crops, as well as welfare and ethical issues particular to animals. Some early research applications of GM to animals had also been subject to considerable scrutiny regarding both animal welfare and the ethical acceptability of the procedures. Technology that was already controversial in a crop context is perceived as even more problematic when applied to sentient organisms, such as farmed livestock (Coles et al. 2015). One of the lessons taken by developers of genome edited animals is that applications with an animal welfare benefit would be expected to be much more acceptable to publics than livestock with production advantages. This is reflected in some of the early applications of genome editing to livestock.

One of the early applications of genome editing is to produce cows without horns (Recombinetics 2016). Dairy cows with horns can harm each other and their caretakers. Therefore it is common practice within the industry to remove the horns from calves by chemical or other means. This removal of horns is an unpleasant procedure, which it would be beneficial to avoid. Cows without horns (known as ‘polled’) exist naturally, but they tend to be beef cattle with much poorer milking ability. Using selective breeding to increase the incidence of the polled gene variant (allele) in the dairy herd would involve a very long process, particularly if milk yield was maintained at the same time. However, this gene variant has been introduced into dairy cows directly using genome editing (Recombinetics 2016). The question remains whether publics and animal welfare advocates perceive the animal welfare benefit to be of sufficient magnitude to justify such a biotechnological intervention. Whereas people working directly with dairy cattle will be aware of the processes involved in dehorning, and the benefits of not having to do so, most publics are likely to be unaware that dehorning processes take place, let alone what these involve in practice. The advantages of polled dairy cattle will not be immediately obvious to those not versed in agricultural practice, and the need for dehorning may be considered shocking.

Increased disease resistance is another area that has been addressed by genome editing, again with perceived animal welfare benefits. Examples include pigs tolerant to a fatal pig disease African Swine Fever (Lillico et al. 2013) and pigs resistance to a serious respiratory and reproductive disease, Porcine Reproductive and Respiratory Syndrome (PRRS) (Whitworth et al. 2016). PRRS is an endemic pig disease in numerous countries and has few treatment possibilities. It negatively affects animal welfare and causes large economic losses. African Swine Fever is a pig disease endemic in Africa that causes up to 100% mortality and for which effective treatments and vaccines are not available. The disease has spread to Russia, and most recently to Eastern Europe, where it is seen as a threat to Western European pig production. Warthogs are found to be tolerant to African Swine Fever and inspired a genome editing method which has introduced the same tolerance alleles into domesticated pigs in an experimental setting (Lillico et al. 2013).

The benefits of other applications of genome editing may be perceived as more ambivalent. Genome editing has been used to produce more muscular animals in a variety of species, including pigs, cattle and sheep, by introducing a change in the myostatin gene (Cyranoski 2015; Proudfoot et al. 2015). The same change is naturally present in some breeds of cattle. In the public mind, muscular appearance often has connotations of poor welfare. Belgian Blue cattle and Pietrain pigs are two breeds that are muscular in appearance and of concern to animal welfare advocates. Belgian Blue cattle have a high incidence of problems when calving, and muscular breeds of pigs have been associated with high mortality rates. The causal mutation is in the myostatin gene in Belgian Blue cattle and the ryanodine receptor/halothane gene in the Pietrain pig. The question arises whether animal welfare advocates are likely to be concerned by physical appearance, the specific genetic change, both, or neither? The experimental introduction reported by Proudfoot et al. (2015) was in the context of very poorly muscled cattle breeds, the resulting offspring physically appearing normal rather than excessively muscled. The myostatin mutation also appears naturally in other breeds such as Highland cattle, without apparent associated welfare problems, so it may be possible to make the case that editing the myostatin gene provides a benefit in specific circumstances. However, the benefit is for production rather than animal welfare. It is less easy to see how a strong case can be made for more muscled pigs if muscling in pigs is associated with poor welfare (even when caused by a different genetic change). Public perceptions associating muscled animals with poor animal welfare can be difficult to shift and applications resulting in increased muscling may therefore prove to be controversial.

As is common with novel technologies, early innovations frequently use material which is readily available, such as already known gene variants. It seems likely that increasingly innovative genome edits will be developed. Suggestions in the scientific literature include: producing offspring of a single sex (e.g. for milk or egg production) (Fan et al. 2013; Tan et al. 2013), improved welfare by avoiding castration (Tan et al. 2013), and the prevention of the production of allergens and prion proteins in animals (Yao et al. 2014; Ni et al. 2014). Genome editing also has applications beyond the production of food animals, notably for research for understanding biological processes, in improved models of human diseases and potentially for production of animals for xenotransplantation. However, medical applications will not be considered further in this paper.

Early applications of genome editing that come to public attention are likely to shape how people think about genome edited animals. These applications are likely to influence what people think genome edited animals are, and the purposes for which they have been developed. The choices made amongst possible applications of genome editing to animals are therefore important. Benefits perceived by scientific or agricultural stakeholders, however, may be sometimes difficult to convey to lay people.

### Is genome editing natural?

An important feature of GM crops is that they are viewed as unnatural by some people. The concept of naturalness is complex, and cannot be considered in detail in this article. However, as a recent report from the UK Nuffield Council on Bioethics (2015) indicates, naturalness should not be totally dismissed as an argument because most people recognise the concept as capturing something valid which should be taken into account. The question remains as to whether any breach of the natural order caused by genome editing would constitute a moral hazard.

Since all genome edited applications rely on laboratory intervention to develop parent animals, at some level, all involve a degree of human artifice. Bruce (2016), reflecting on whether genome editing is ‘unnatural’ or not from a moral reasoning perspective, suggests that it is important to consider the nature of the change brought about by genome editing. If selective breeding (genetic selection) is considered natural, and if the functional change brought about by genome editing could have been achieved by selective breeding, then it could be argued that there is little reason to object to genome editing in principle. An example is producing polled dairy cows. In this case, the same DNA sequence exists in nature and in the context of the same species. The question remains as to why it would be seen as natural to undertake selective breeding for a specific aim, but unnatural to do the same in a laboratory using genome editing.

Genome editing may produce changes that are not known to exist naturally in that species. But if these could reasonably have occurred naturally, even if they remained unrecognised by livestock breeders, it could be argued that these changes are also ‘natural’. The editing of resistance to PRRS into pigs might fit this category, as it is a mutational knock-out of gene function (Burkard et al. 2017).

It is easier to categorise as unnatural those applications where the resulting animal is unlikely to have been achieved by normal mutation, for example producing offspring of a single sex. It is therefore possible to advance arguments that some genome edited animals are more unnatural than others. There is a similarity here with GM crops. It is possible to breed crop plants resistant to specific herbicides using traditional breeding methods, although the genetic basis for the resistance may vary between GM crops and non-GM crops. Equally, it is possible to produce novel applications by genetic modification, as exemplified both in GM crops and GM animals. It is notable that the few current commercial applications of genetic modification to animals involve the production of pharmaceutical proteins (in milk or eggs). These traits are unnatural, yet have utility to humans. Clearly, just because something is considered unnatural, does not necessarily mean it is undesirable. Similarly, some natural mutations may be judged undesirable.

Although philosophers, theologians, ethicists and scientists may contest the various arguments as to what constitutes naturalness, lay publics may conceive of naturalness on a more intuitive basis. Such perception of unnaturalness can be very difficult to argue against.

### Is genome editing genetic modification?

The next lesson learned from experience with GM crops is the controversial nature of moving genes across species. An emphasis found in many scientific papers is that genome editing does not necessarily mean transgenic. Furthermore there is a tendency to equate transgenic with genetically modified. An example can be found in the report of genome editing for resistance to PRRS (Whitworth et al. 2016) which explicitly states that the resultant pigs are not transgenic and therefore not genetically modified. This assertion is controversial, as the concept of what is genetic modification varies both legally and, more particularly, socially.

If the term ‘genetically modified’ is viewed as synonymous with ‘transgenic’, the implication is that since genome editing does not require the transfer of DNA from one species to another, then genome edited organisms, including livestock, are not genetically modified. This semantic difference may be attractive to proponents of genome edited animals, but it is unlikely to convince everyone, given the technology has developed in the context of genetic modification, and can be viewed as evolving from those techniques. The mere fact that genome editing comes from the same ‘stable’ as genetic modification can be sufficient to give it the same association.

On the other hand genome editing could also be viewed as distinct from genetic modification. Genome editing involves harnessing the cell’s natural repair mechanisms to induce a specific change at a precise location in the genome. This type of change could have happened by natural mutation. Genome editing could therefore be viewed as ‘induced mutation’, with parallels to using radiation to induce mutations in plants (although the latter is of course, random, and would be unacceptable if applied to animals). However, genome editing is not perfectly controlled due to ‘off-site’ effects, although recent research suggest that off-site effects can be controlled to a large extent (Tan et al. 2016) and gene sequencing could be used to detect presence or absence of off-site effects.

An alternative framing for genome editing could be Marker Assisted Selection. Marker Assisted selection relies on taking biological samples from animals and analysing these for the presence or absence of genetic markers associated with specific traits. Some of these markers (such as the ryanodine receptor/halothane gene) are single nucleotide changes. Marker Assisted Selection has been viewed more favourably than genetic modification by some stakeholder groups. For example Greenpeace has advocated Marker Assisted Selection in crop plants, on the basis of (1) respecting species barriers; (2) increasing the efficiency of traditional breeding without replacing it; and (3) treating genomes as coherent entities rather than transferring isolated gene sequences (Greenpeace International 2009).

Genome editing has some similarities with Marker Assisted Selection. It does not necessarily transgress species barriers and arguably treats the genome as a coherent entity in so far as any change involved is mimicking a natural mutation. However, genome editing does require a deliberate intervention that is not possible without biotechnological capability that is outside traditional farming practice. On the other hand, ‘traditional’ farming practice has already been supplemented in many cases by centralised specialist procedures that lie outside the traditional farming sphere. These include central performance testing, semen distribution, data collection, sophisticated statistical analysis, and in the case of marker assisted selection above, biological sampling and analysis. Selective breeding is based on phenotypes, and separation of genotype and environment using statistical methods. This has further developed into genomic selection, which involves the use of tens of thousands of genetic markers across the genome rather than relying on individual markers. Genomic selection has been made possible through increases in computing power, easier and cheaper availability of genotype information, and developments in statistical techniques. Genomic selection is being applied commercially in, for example, dairy cattle. Since the physical animal has already been ‘converted’ into data, and these data are increasingly converted to genetic information though genome selection, it seems a short step to begin to manipulate these DNA changes in purposeful ways. Genome editing and genome selection may even become integrated, as suggested by Jenko et al. (2015). Thus the boundary between current practice of genomic selection and genome editing may become increasingly blurred.

What then is genome editing? Is it genetic modification? Induced mutation? Marker Assisted Selection? Or genome selection? Or something else? From the above discussion, these definitions cannot be answered merely from a scientific perspective, because genetic modification has become as much a social concept as a scientific concept.

The nuances that these descriptions capture for a scientific audience can be difficult to understand for those outside this specialist sphere. Evidence from psychology suggests that when confronted by a new phenomenon, people tend to use a fast way of thinking in order to make sense of that phenomenon (e.g. Forgas 2001; Kahneman 2011). This way of thinking seeks to identify what the new phenomenon is similar to, rather than carefully evaluating all the details. The results of this type of thinking can seem illogical to those versed in the details of a technology. In this way, for example, for a lay audience, genome editing could easily be rolled in with genetic modification, cloning and tasteless tomatoes in supermarkets.

It would of course be advantageous for developers of genome edited animals if they were able to avoid the onerous regulations associated with genetic modification, so the argument that genome edited animals are not genetically modified because they are not transgenic, is attractive. In this next section I consider the interactions between genome editing and regulation.

## GM regulation and genome editing

Two aspects of regulation are considered in this section. The first is whether genome edited animals should be subject to the same regulations as GM animals. Here, I will primarily take an EU perspective, given the variety of regulatory approaches around the world. In this context, the main question is whether genome editing counts as genetic modification and is therefore subject to GM regulations? The second reflection is on the impacts that GM crop regulation has had on industry structures and the relative competitiveness of different sectors.

### GM regulation and genome editing

GM salmon (AquAdvantage™) modified for faster growth, has been at the forefront, worldwide, of the regulatory process for GM animals for food production (Van Eenennaam and Muir 2011). However, the approval process for AquAdvantage™ salmon (which used classical GM technology) was extremely onerous, and supporters of genome edited animals advocate much simpler, or even no regulatory constraints for genome edited animals.

The EU regulatory system was developed in the context of GM crops, is process based and depends on a specific definition of genetic modification. The EU defines a genetically modified organism as:

an organism, with the exception of human beings, in which the genetic material has been altered in a way that does not occur naturally by mating and/or natural recombination (Directive 2001/18/EC, Article 2(2))

A key question is therefore whether genome editing legally constitutes genetic modification in the EU or not? While the EU continues to debate this issue, some preliminary decisions have been made elsewhere.

A number of genome edited organisms have been determined to fall outside the regulatory purview of the USDA’s Animal and Plant Health Inspection Service (APHIS) e.g. mushrooms edited using CRISPR-Cas9 to reduce browning (Waltz 2016). However, regulatory oversight in the USA is not triggered by whether the process involves genetic modification. Rather the regulatory triggers for GM organisms are determined by the underlying laws and regulatory authorities of each U.S. regulatory agency and depends on the nature of the product. Similarly genome edited crop plants, such as canola, have gained Plant Novel Trait approval in Canada (Cibus 2014), where again regulation is not triggered by whether the process involves genetic modification. Within the EU there continues to be debate as to the regulatory status of plants produced by genome editing, with a variety of different interpretations being put forward (European Parliament 2016). The Swedish Board of Agriculture has concluded that some applications of CRISPR-Cas9 do not fall under the EU definition of a Genetically Modified Organism (GMO) and the German Federal Office for Consumer Protection and Food Safety has come to a similar conclusion (European Parliament 2016).

On January 18, 2017, the U.S. Food and Drug Administration (2017) released for public comment their Draft Guidance on the Regulation of Intentionally Altered Genomic DNA in Animals. The draft guidance recommends that genome edited animals should be regulated in a manner similar to that used by the agency to regulate GM animals. At the time of writing, the draft guidance was still in the public comment period.

The narrow consideration of whether genome editing is a form of genetic modification or not, does not of course address the questions of what is the purpose of regulation. From the perspective of different genetic changes, Van Eenennaam and Young (2015), cogently argue that current regulatory regimes fail to reflect the scientific evaluation of risks from different applications. In practice, however, objections to developments may be on grounds other than risk, but are nevertheless expressed in terms of risk, as the only effective way of stopping a development that is unwanted (Tait 2001). Examples of such objections with respect to GM crops include excessive power of commercial companies over food systems and excessive industrialisation of agricultural practices. There is no clear mechanism where such objections can be considered, and hence arguments against developments tend to focus on risk.

In the context of GM crops, suggestions have been made to establish ways of facilitating more open debate around the non-science based evaluations applied to policy decisions and making the basis for policy decisions more explicit (Devos et al. 2013). Areas suggested for more open debate by Devos et al. include clarifying policy objectives, determining what constitutes environmental (and by analogy animal welfare) harm, making explicit the normative basis of risk assessments (what level of risk is acceptable and who should bear the risk), and weighing harms against benefits.

If genome editing is to be harnessed for animal welfare, environmental or social benefit then some kind of regulatory or other ‘carrot’ to drive in these directions, would provide the greatest opportunity for such developments, rather than merely relying on markets. An example from another industry sector, is the U.S. Food Quality Protection Act that gives preference to pesticides which provide environmental benefits over current products. Some jurisdictions in Europe already include requirements for social benefit in the context of regulatory approval of biotechnology-derived animals. For example, Norway requires GMOs to benefit society and be ethically justifiable as well as not harmful (Library of Congress 2014). In Denmark cloning and genetic modification of animals is restricted to applications benefitting human health and the environment (NordForsk 2016). In the Netherlands, genetic modification of animals for food purposes needs a license requiring the product to serve a public interest and have no overriding ethical objections (Government of the Netherlands 2016). However, these are all examples of regulatory ‘sticks’, designed to prevent applications, rather than ‘carrots’ designed to promote applications considered to be particularly beneficial.

Regulatory regimes for genome edited livestock remain unclear, however, attempting to avoid all regulation could stimulate accusations that genome editing is trying to avoid public debate. By hiding the nature of genome editing developments people may be prevented from having any choice in the matter. Even if the regulatory regime for GM organisms is judged excessive for genome edited organisms, some kind of opportunity for public debate, and provision of methods for identifying products from genome edited animals seems necessary. For example, setting standards for genome edited animals, perhaps drawing on the experiences of regulation of medical devices. Given that producing genome edited animals will require serious investment, it is likely that developers will seek to recoup costs by selling the resulting breeding animals at a premium, and therefore will not seek to ‘hide’ the fact that the animal is a result of genome editing. Nevertheless, such public perceptions may persist.

### Interaction between regulation and company strategies

Turning now to the second aspect of regulation, that of interactions between regulation, company strategies, relative competitiveness and innovation trajectories. Regulation arguable impacts are not just on the safety and efficacy of innovations but also on company strategies and the types of firm likely to be successful (Mittra et al.^2011^). Tait (2007) suggests that adopting a high regulatory burden for GM crops meant that large, multinational agrochemical companies were favoured over traditional crop seed producing firms. The agrochemical companies had both the funds and the expertise to deal with complex regulatory requirements, which the seed companies did not have. If genome edited livestock will be required to comply with the same regulatory standards as GM crops, then again companies with the resources to cope with such regulatory burdens are likely to be favoured.

Analysis of agrochemical company strategies (Tait 2007; Tait and Chataway 2007) suggested that genetic modification of crops was disruptive innovation for this industry sector. Agrochemical companies used to innovation based on small chemical molecules needed novel innovation pathways for producing GM crops. The companies could not continue using existing innovation pathways, but instead required path-breaking innovation involving new areas of research and new markets. At early stages of development of GM crops, agrochemical companies used various strategies to deal with this disruptive innovation, including acquiring seed companies (Monsanto and Dupont), collaborating with seed companies (AgrEvo, Zeneca, Novartis, Rhine Poulenc and Dow) or waiting to observe developments (BASF and Bayer) (Mittra et al. 2011).

In comparison, GM crops would have been incremental innovation for traditional seed producing companies. They could have continued their current innovation trajectories and gained competitive advantage through existing business practices (Tait 2007). Tait (2007) further suggests that had seed companies produced GM crops, the public outcome for GM crops may have been very different. The high standard of evidence required to satisfy regulatory requirements, however, has meant that regulation has effectively acted as a barrier to entry for smaller traditional seed producing firms (Tait 2007).

Research papers reporting the achievement of a few genome edited animals are important accounts of scientific advancements. However, it is not enough just to produce three or four genome edited animals for the application to become commercially viable. To produce a commercially viable product, the number of animals needs to be increased, and the animals and their products accepted by a range of stakeholders, including animal breeders, farmers, vets and supermarket chains, as well as successfully negotiate any regulatory barriers. A plausible route to market is needed. In the case of genome edited animals, current developments appear to include those where a plausible route to market is developing. For example a major, global livestock breeding company, Genus plc, has announced it has an exclusive licence to use genome editing to commercially produce pigs resistant to PRRS (Genus 2015).

The international livestock breeding sector is well organised, particularly when considering chickens, pigs and dairy cows, although with some heterogeneity. A number of global breeding companies dominate the sector. Specialist breeding companies undertake genetic selection to produce the next generation of livestock, multiply this stock (and often produce cross breeds) and sell to commercial farmers. Unlike crops, where the whole population can be rapidly replaced, introducing genome editing into a livestock population is a much more complex and time consuming process.

Although the entry of one major breeding company into genome editing is noted, the breeding company model is not the only one in existence in the supply of breeding stock, as state intervention in livestock breeding has been common in many European countries. As with other industry sectors, there is evidence that the breeding sector has been shaped in part by regulation. In France, for example, state intervention, and then the withdrawal of the state from the breeding sector has had a profound impact on the commercial stakeholders (Hannachi and Tichit 2016). The 1966 Livestock Act, introduced to encourage the use of Artificial Insemination, resulted in the creation of monopoly breeder co-operatives in different regions, and the loss of influence by key individual breeders. The later Agricultural Orientation Law (2006) resulted in loss of state intervention, which together with technological changes due to the introduction of genomic selection, allowed the entry of multinational companies into the breeding sector. Hannachi and Tichit argue the result is that cattle breeding in France has moved from being a collective, collaborative exercise, to one that is practiced individually and characterised by competition (Hannachi and Tichit 2016). This is an example from the livestock production sector of the influence that regulation can play on subsequent industry development.

The nature of the genome editing application may also have an impact on the innovation trajectory and its impact on different parts of the livestock production system. Applications such as polledness are likely to be path-dependent for farms, although suppliers of materials to dehorn animals may see their markets decline and may need to identify novel markets. A cow which does not develop horns is still just a cow and the basic farm business model is not disrupted by its lack of horns. The hornlessness reflects improved animal welfare, labour savings in avoiding an unpleasant task and economic savings in materials and labour. However, other potential genome edited products have the potential for more disruptive impacts.

The ability to avoid the need for castrating animals could have stronger animal welfare benefits, and additional benefits in reduced labour requirements, as well as better performance from animals that do not suffer a growth-check from the procedure. The process may also be potentially disruptive, for example if this allows novel meat products to be developed as pigs could be slaughtered at heavier weights if the taste taint from males is absent. An application which provides the ability of only producing commercially useful animals of the desirable sex has the potential to be disruptive in ways which are hard to predict, as reproduction could be limited to specific farms. There may need to be novel social or institutional arrangements that allow maintenance of the parent animals of both sexes, with consequent impacts on costs of production and distribution of control and ownership of breeding stock.

Public acceptability of such radical applications also need to be questioned. Few such data exist currently, although for example an on-line survey in the Netherlands (Gremmen and Blok 2016) asked respondents to compare the acceptability of a number of different options for dealing with unwanted male chicks in egg production. The respondents in this survey considered that genetically modifying chickens so that eggs with male chicks could be identified by green fluorescence, was more acceptable than taking biological samples from non-genetically modified eggs to identify males by laboratory means. Although this is a small example, and may not be reflected in broader acceptability of using genome editing to alter sex ratios, it does indicate the importance of examining public attitudes rather than presuming them.

Within the livestock production systems, different stakeholders are likely to be affected in different ways. Genome edited animals are sold to farmers not consumers. For farmers, genome edited animals are likely to be incremental innovations, whose management is path dependent. The ‘product’ is after all, a cow or pig, like any other. There may be additional recording and identification requirements if labelling and co-existence requirements are similar to those for GM crops in the EU. However, the demands for recording individual animals and animal movements in the EU are already considerable.

For breeders without laboratory science expertise, developing genome edited animals is likely to be path breaking, requiring the acquisition of new skills. However, larger companies in this sector have a very high degree of technical absorptive capacity. Distribution of genome edited animals is likely to be path dependent as the methods of distribution depend on the physical material. However, meeting the requirements of intellectual property regimes allied to genome edited animals may prove to be disruptive. Hitherto the intellectual property in livestock breeding has been primarily protected by secrecy and use of cross-breeding. Genome edited animals could be path-dependent for meat processors and retailers, unless there are consumer requirements for labelling or campaigns against such products, in which case genome edited animals could require new business practices to be developed. One sector that could easily be affected, and for whom this is potentially a path-breaking development, is the veterinary sector.

## Veterinary sector

Disease control is an initial focus for several genome editing projects, the examples of African Swine Fever and PRRS were given above. These diseases have few or no alternative treatments and also pose a significant economic threat to pig production. If genome edited disease resistant or tolerant animals are to be widely adopted, disease resistance would need to be incorporated into overall disease management strategies at the individual farm, or even national, level.

Genetic disease resistance is intended to be used in disease prevention. However, based on a historical analysis of UK veterinary practice, Woods (2013) argues that vets have used the rhetoric of disease prevention, but found it difficult to deliver in practice for a variety of reasons. They have instead maintained an approach that focusses on treatments. Woods suggests that for effective prevention, an appropriate combination of veterinary, disease, farming and political drivers are required, all of which promote disease prevention. An important step is to engage the veterinary profession and other appropriate stakeholders to identify the drivers necessary for genome edited animals to be harnessed for animal health improvement. Some reflections on issues worth exploring are given in the following paragraphs.

Genome edited animals are unlikely to be adopted by all farmers. Therefore, it is likely that disease resistant or tolerant (depending on the nature of the edit), and disease susceptible animals would co-exist in commercial populations. Understanding the epidemiological implications (if any) of introducing a novel resistance trait to some animals, could be one of the questions being addressed. Such an examination could also uncover veterinarians’ perceptions of genome edited disease resistant animals and the potential impact of such animals on their work.

Veterinarians are responsible for implementing disease control measures, particularly where there are specific legal requirements for doing so. This would be the case should there be a suspected emergence of African Swine Fever in the UK, for example. Genome editing thus could have much wider implications, because the suspicion that a notifiable disease of international significance is present can cause major disruption to international trade. The potential implications of genome editing for disease resistance in the event of any future outbreak of such diseases could therefore be explored by a range of stakeholders.

Part of the current European policy landscape is that the costs of exotic disease prevention should be shared between government and farmers, as opposed to governments bearing all the costs of exotic disease prevention. Could farmers be encouraged to keep disease resistant livestock by being given a greater compensation if they do so, if an exotic disease outbreak were to occur? Farmer behaviour related to livestock disease is complex and context specific (Barnes et al. 2015). But if farmers were to be offered higher compensation for adopting disease resistant animals, then the resistance would need to be attested to in some way. This suggests the need for some kind of standards certification, which would identify animals with appropriate disease resistance characteristics.

While these considerations are not unique to genome edited animals—some of the same could apply to selective breeding for disease resistance—addressing these types of questions could be important when considering commercial realities of genome edited animals. Taking account of the way in which veterinarians’ practices interact with genome edited animals in different circumstances would test interactions among different parts of a livestock production system.

## The future of livestock production

GM crops were introduced in the late 1990s at a time when the use of chemicals in agriculture had become controversial. Advocating the introduction of crops that promoted the use of a pesticide or herbicide (although environmentally more benign than some others) in this context provided a ready basis for public resistance. There are currently many different (and often competing) visions for the future of livestock agriculture that set the wider context for genome editing. For example, the sector has come under intense pressure from environmental considerations, particularly the need to reduce greenhouse gas emissions, while at the same time responding to the imperative to increase livestock production to feed burgeoning populations (Godfrey et al. 2010). A recent policy paper from a European livestock industry perspective starts from the premise that the livestock sector has been identified as the world’s largest user of natural resources (Knowledge for Innovation 2015). This policy paper calls for innovation in the livestock sector that is environmentally sound, socially responsible and economically viable. The extent to which genome edited animals can contribute to visions of the future of agriculture will impact on future trajectories. Many different future visions exist. Two contrasting visions are provided here, as exemplars.

One vision of the future of agriculture focusses on the current, conventional, global systems of livestock production as typifying such a future. Typically, farms would continue to provide livestock products for global markets, with a strong emphasis on the efficiency of production. This vision would be likely to embrace application of various technologies such as exploiting information technology and the use of rapid environmental and biological diagnostics. Within this vision, measures to improve efficiency would be valued and might encompass a range of metrics, such as carbon footprints, the efficiency of use of animal feed for growth, and biological measures such as reproductive efficiency. Contributing to these efficiency measures would be viewed as contributing to delivering environmentally sound, socially responsible and economically viable livestock production. In such a context, genome edited animals could be seen as consistent with these aspirations.

In contrast to the global production system, there are a group of alternative livestock production systems which aspire to transform the global production system. These alternative production systems are diverse, and include for example, Community Supported Agriculture, urban agriculture, local production, and marketing through farmers’ markets and food hubs. They have developed partly as a way of resisting what is perceived as the dysfunctional current global production system. A key criticism of the global system is the way in which consumers have become separated from producers. Rather than emphasise metrics of efficiency, these alternative production systems stress equitable relationships between consumers and producers, and between livestock and human beings. Traditional forms of knowledge and use of breeds adapted to local conditions are emphasised. New knowledge is by no means rejected but the role that genome edited animals could play in such a scenario is more questionable.

Genome editing for improved adaption to local conditions might be compatible with the aims of ‘alternative’ systems, for example if animals were edited to be more heat tolerant. However, genome editing may be rejected in principle by advocates of this future vision, on the grounds of reflecting an excessively instrumental relationship between humans and livestock. The emphasis in this future vision on resistance to global livestock production systems may also militate against genome editing, if genome editing is perceived to be associated with those global systems.

## Conclusions: key lessons for genome edited livestock

Genome editing promises the possibility of producing commercial livestock carrying precise genetic changes, which are difficult or impossible to achieve with selective breeding. Ultimately, the future of these developments will depend on political decisions, regulatory requirements and public acceptability, as well as technological capabilities. This paper has sought to draw on the experiences of the introduction of GM crops to examine the development of genome edited livestock. In particular, this paper extends the focus on science and regulation, to considering the wider context of livestock production systems.

Early applications of genome editing to livestock have taken into account the need to provide publicly recognised benefits, as a lesson learned from the production-focus of early GM crops applications. Initial applications have mainly focussed on welfare and disease resistance traits, although more profound changes to animal physiology have also been suggested, and may be realised in future. One of the challenges of meeting public aspirations for welfare benefit is the difficulty of agreeing on what is a welfare benefit. What may seem beneficial, or at least neutral, in terms of animal welfare for those familiar with livestock production, can be more controversial for others. Public views on more profound changes to physiology, e.g. altering sex ratios, are as yet, largely unexamined. In general attitudes are likely to be influenced by the particular reason given for the application, how beneficial or risky it is considered to be, and specific context of application and the alternatives available.

Early applications of genome editing in livestock have also focused on creating gene variants that exist in the same, or similar species, and seeking to avoid crossing species boundaries. Given the tendency among some people to conflate genetic modification with transgenesis, genome editing might be thought to provide an opportunity to avoid the regulatory impasse that in many cases has been a feature of GM animals. However, avoiding regulation for genome edited animals on the basis that they do not involve crossing species barriers would restrict applications of genome editing only to those that meet this requirement, and could discourage many other developments. At the other extreme, avoiding any kind of regulation for genome edited animals could also easily result in a public back-lash. Some publics may see unregulated adoption of the technology as a way to introduce (by stealth) practices that they think are cruel or unnecessary. A carefully nuanced mechanism for identifying and regulating genome edited animals seems essential.

Genome editing applied to livestock production is not just a technical fix that can be effortlessly adopted without consequences to the wider production system. Stakeholders in the wider industry may react in ways that perturb current practices. These perturbations can be application specific or context specific, and may be difficult to predict without engagement with a range of appropriate stakeholders. In the case of GM crops, regulatory requirements and company strategies led to multinational agrochemical companies being favoured over crop seed producers. Early indications for genome edited livestock are, that at least one global breeding company is willing to adopt genome editing and has the capacity to deliver genome edited livestock to farmers. Adoption of genome editing is consistent with the expertise present in this company. It is not clear what impact genome editing will have on other industry stakeholders, such as smaller breeding organisations or nationally supported breeding co-operatives.

Genome edited specific disease resistant livestock can have implications on management of disease, in particular diseases that are exotic to the particular jurisdiction and which have the potential to disrupt international trade. On the other hand, disease resistant livestock could be incorporated as part of a package of actions aimed at better prevention of disease.

The wider context of livestock production is also important to consider. Pressure is increasing on the sector to reduce its environmental impact, while maintaining productivity. Whether genome editing can contribute to these aspirations remains to be seen.

Public support for genome edited livestock is essential for the promised products to gain wide market penetration. Frivolous, or controversial applications raising public disquiet have the potential to make it very difficult for future genome edited livestock applications to be socially accepted. Competition to be the first on the market needs to be tempered by consideration of the wider future of the technology. Focussing on technology and its regulatory implications is important but more attention needs to be paid to interactions among stakeholders to better understand how genome edited animals could impact the broader livestock production sector.
